# Relationship between the Effectiveness of Arthrocentesis under Sufficient Pressure and Conditions of the Temporomandibular Joint

**DOI:** 10.5402/2011/376475

**Published:** 2011-05-31

**Authors:** Shinya Yura, Kazuhiro Ooi, Yuri Izumiyama

**Affiliations:** Department of Oral and Maxillofacial Surgery, Tonami General Hospital, 1-61, Shintomi-cho, Tonami-city, Toyama-ken 939-1395, Japan

## Abstract

*Background*. The purpose of this study is to investigate the conditions of the temporomandibular joint relative to the effectiveness of an arthrocentesis-like enforced manipulation technique followed by irrigation under high pressure in patients with closed lock. *Methods*. We performed arthroscopic examination and manipulation followed by irrigation as the initial treatment in 50 joints with closed lock. Relationship between the effectiveness of the procedure and conditions of the temporomandibular joint was statistically analyzed using multiple regression analysis. *Results*. Significant inverse correlations were found between the extent of improvement in maximum mouth opening after treatment and the initial maximum opening before treatment. There were no significant correlations between improvement of joint pain at mouth opening and in biting and conditions of the temporomandibular joint. *Conclusions*. Pathologic conditions of the temporomandibular joint did not have an influence on the efficacy of the technique. This result suggests that this procedure has wider application than conventional arthrocentesis.

## 1. Introduction

Arthrocentesis is an easy, minimally invasive, and highly efficient procedure to decrease joint pain and increase the range of mouth opening in patients with closed lock of the temporomandibular joint (TMJ). This can be performed under local anesthesia in the outpatient clinic. The effectiveness of conventional arthrocentesis, irrigation under low-pressure with an elevated infusion bag or syringe, has been reported. In those studies [1–3], low pressure arthrocentesis was effective for treating acute closed lock without severe joint pain or bone change, but it was not always effective for treating chronic closed lock with osteoarthritis, synovitis, or adhesions in the upper joint space.

 Previously, we reported the efficacy of a modified procedure with an arthrocentesis-like enforced manipulation technique followed by irrigation under high pressure (40 KPa). In that study [4, 5], no significant correlation was found between the effectiveness of the procedure and the pathologic conditions of the TMJ. The result indicates that the application of this procedure is wider than that of arthrocentesis under low pressure. However, the presence of disc perforation was not included in the pathologic conditions and factors related to the improvement of the joint pain in biting were not investigated. Therefore, we further studied the effectiveness of this procedure and statistically analyzed the correlation between the effectiveness of the procedure, including improvement of the joint pain in biting, and the pathologic conditions of the TMJ, including disc perforation.

## 2. Patients and Methods

### 2.1. Patients

Fifty patients (50 joints) with chronic closed lock of the TMJ who underwent arthroscopic examination and arthrocentesis under sufficient pressure at Tonami General Hospital, Tonami, Japan, from 2004 to 2008, were included in this study. The patients were 5 males and 45 females, ranging in age from 12 to 71 years, with a median age of 44 years. Duration of symptoms of the patients ranged from 3 to 48 months, with a median of 4 months. All of the patients were found to have anterior disc displacement without reduction, evident by magnetic resonance imaging.

### 2.2. Magnetic Resonance Imaging

Magnetic resonance imaging was performed using a 1.5-T imager (Signa, General Electric, Hino, Japan) with a dual 3-inch surface coil. T1-weighted and fat-saturated T2-weighted images were obtained in both the sagittal and coronal planes. The transaction plane was scanned to find the long axis of the condyle; the sagittal plane was considered to be perpendicular to this long axis with 3-mm section thickness and the coronal plane was considered to be parallel to the long axis with 3-mm section thickness. The degrees of disc shape and bone change were examined with these sequences.

### 2.3. Arthroscopic Examination and Arthrocentesis

**Figure 1:**
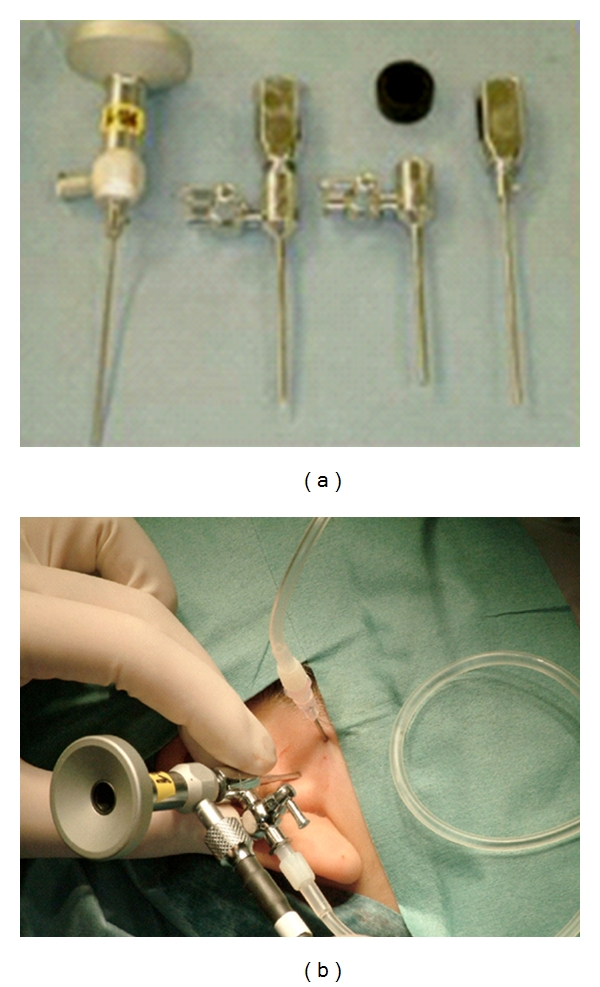
A 1.2-mm diameter ultra-thin arthroscope.

Arthroscopic examination and arthrocentesis were performed by the same specialist within 2 weeks after the magnetic resonance imaging, according to the technique of Yura et al. [4, 5]. A 1.2-mm diameter ultra-thin arthroscope (DRK-21, Shinko Optical Co., Ltd., Tokyo, Japan) was used in the examination (Figure 1). The absence and degrees of synovitis, cartilage changes, adhesion, and disc perforation in the upper joint compartment were observed using the arthroscope. Types of these pathologic conditions [5] are shown in Table 1.

**Figure 2:**
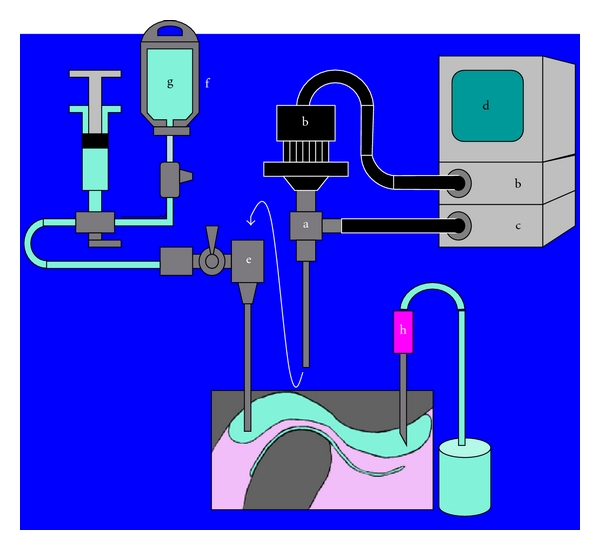
Arthroscopic surgery. Shown are the arthroscope (a), camera (b), light source (e) monitor (d), trocar (e), infusion accelerator for a blood bag (f), saline solution (g), and 18-gauge needle (h).

A schematic image of arthroscopic examination and arthrocentesis is shown in Figure 2. Saline solution was injected to widen the upper joint space. An 18-gauge needle was inserted into the anterior recess of the space and a trocar for an arthroscope was inserted into the posterior recess of the space. The intracapsular pathologic conditions were observed before irrigation. During irrigation, the arthroscope was removed from the trocar and the tube for irrigation was connected to the trocar. High pressure was then added to the space using the infusion accelerator for a blood bag (maximum pressure exerted, 40 KPa). In 5 min, 300 mL of saline solution was injected into the joint space. The needle was withdrawn after the injection of 10 mg prednisolone and then enforced manipulation with a range of mouth opening of 40 mm or more was performed. After arthrocentesis, the patients continued opening protrusive and lateral excursive exercises for 2 months. 

### 2.4. Methods

The patients were examined before arthrocentesis and every 2 weeks after arthrocentesis for 8 weeks. Their maximum mouth opening (MMO), joint pain at mouth opening, and joint pain in biting, measured on a visual analog scale (0 to 100), were evaluated. The effectiveness of the arthrocentesis was evaluated from any range of increase in MMO (in mm) and improvement rate of joint pain (percentage) after the procedure. With respect to conditions of the patients before arthrocentesis, we examined sex, age, duration of symptoms, MMO, joint pain, and degrees of disc deformity, bone change, synovitis, cartilage change, adhesion, and disc perforation.

The Mann-Whitney's *U* test and Spearman's rank correlation coefficient were used for analysis of the correlation between the effectiveness of the procedure 1 month after surgery and conditions of the patients. Furthermore, these factors were statistically analyzed by multiple regression analysis (StatView ver. 5, SAS Institute).

## 3. Results

### 3.1. Effectiveness of Arthrocentesis under Sufficient Pressure

**Figure 3:**
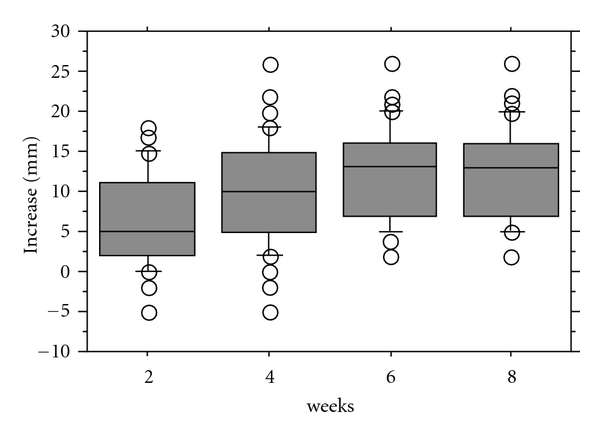
Increase in maximum mouth opening.

**Figure 4:**
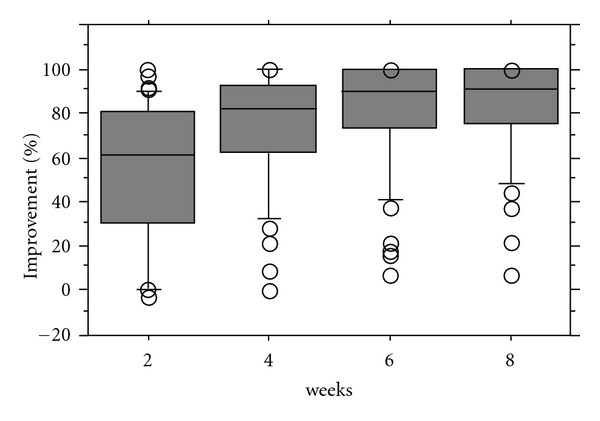
Improvement of joint pain at maximum mouth opening.

**Figure 5:**
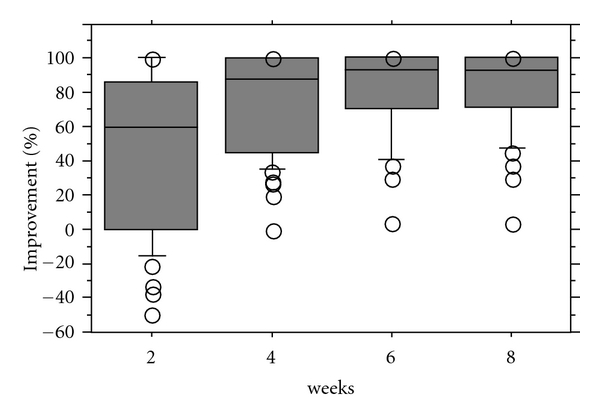
Improvement of joint pain in biting.

Eight weeks after arthrocentesis, the MMO of the patients improved a median of 13 mm (range, 2 to 26 mm) (Figure 3), the joint pain at mouth opening improved a median of 90.85% (range, 7.1% to 100%) (Figure 4), and the joint pain in biting improved a median of 94.35% (range, 4.1% to 100%) (Figure 5). 

### 3.2. Factors Related to the Effectiveness of Arthrocentesis under Sufficient Pressure

The correlations (*P* value) between the effectiveness of arthrocentesis 1 month after surgery and conditions of the patients are shown in Table 2. Significant inverse correlations were found between the extent of improvement in maximum mouth opening 1 month after the procedure and the initial maximum opening before the procedure. The coefficient of determination *R* ^2^ = .450 was analyzed by multiple regression analysis. There were no significant correlations between improvement of joint pain at mouth opening and in biting and conditions of the TMJ (*R* ^2^ = .191 in joint pain at mouth opening, *R* ^2^ = .170 in joint pain in biting).

## 4. Discussion

In previous reports [1–3], the effectiveness of arthrocentesis under low pressure in patients with severe preoperative joint pain, synovitis, adhesions, or bone changes was significantly lower than that in patients without these conditions. We previously reported on a technique and its efficacy in performing arthrocentesis under sufficient pressure [4, 5]. Pathologic conditions of the TMJ, such as disc deformity bone changes, synovitis, cartilage changes, and adhesions, did not influence the improvement in MMO and joint pain at mouth opening [5]. The present study showed that no significant correlation was found between the efficacy of the procedure, including improvement of the joint pain in biting, and pathologic conditions of the TMJ, including disc perforation. These results support and extend those of our prior investigation [5]. This procedure can be performed under local anesthesia in the outpatient clinic. In the institution without an ultra-thin arthroscope, an 18-gauge needle is inserted instead of a trocar for the arthroscope into the posterior recess of the joint space. This is an easy, minimally invasive, and highly efficient one in patients with persistent closed lock. The application of this procedure is wider than that of arthrocentesis under low pressure, and the technique should be introduced widely [6].

A significant inverse correlation was found between the increase in the posttreatment MMO measurement and the initial pretreatment MMO measurement. In this study, the correlation supported the effectiveness of the procedure, which was evaluated by the increase in MMO. Adhesions did not have an influence on the efficacy of arthrocentesis under sufficient pressure, because the technique could remove adhesions and widen the joint space [4]. On the other hand, although hyperplasia of the synovium and bone absorption of the condyle may not improve immediately after irrigation, why the effectiveness of treatment was influenced by a difference in hydraulic pressure during irrigation is not clear. Although the mechanism is uncertain, the reason for this may be that substances related to synovitis and bone changes, such as matrix metalloproteinases that are strongly connected to extracellular matrices, are thoroughly removed by irrigation under high pressure.

In the correlation between the improvement of MMO and conditions of the patients, *R* ^2^ = .450 was analyzed by multiple regression analysis. The result indicated that the correlation could be somewhat explained. Alternatively, with respect to the factors related to improvement of joint pain, *R* ^2^ < .20 was analyzed. The result indicated that the presence of other factors strongly related to the effectiveness of the procedure.

## 5. Conclusion

We studied the effectiveness of arthrocentesis under sufficient pressure and statistically analyzed the correlation between the effectiveness of the procedure. Pathologic conditions of the TMJ did not influence the improvement in MMO, joint pain at mouth opening and joint pain in biting. This procedure can be performed under local anesthesia in the outpatient clinic. This is an easy, minimally invasive, and highly efficient one in patients with persistent closed lock. The application of this procedure is wider than that of arthrocentesis under low pressure, and the technique should be introduced widely. 

## Figures and Tables

**Table 1:** Classification of MR images and arthroscopic finding.

Classification	No. of joints
Types of disc shape on MR images	

Normal: biconcave	20
Mild: enlargement of the posterior band	12
Severe: biconvex, folding, or other pronounced deformity	18

Types of bone changes on MR images	

Normal: normal cortical bone without erosions	31
Mild: localized erosions	9
Severe: extensive erosions with severe absorption	10

Types of synovitis on arthroscopic findings	

Normal: normal synovial lining with a fine network of capillaries	0
Mild: localized capillary hyperemia	10
Severe: extensive capillary hyperemia and hyperplasia	40

Types of cartilage changes on arthroscopic findings	

Normal: smooth surface of articular fibrocartilage	11
Mild: localized superficail fibrillation in the articular cartilage	18
Severe: extensive deep fibrillation and exposure of subchondral bone	21

Types of adhesions on arthroscopic findings	

Normal: no adhesions	14
Mild: localized bandkike or membrane-type adhesions	9
Severe: extensive wall-like adhesions	17

Presence of disc perforation	

Negative	7
Positive	43

**Table 2:** Relationship between effectiveness of arthrocentesis and conditions of the temporomandibular joint (TMJ).

	Effectiveness of arthrocentesis (*P* value)		
Conditions of the TMJ	MMO^†^	Joint pain at MMO^‡^	Joint pain in biting^§^
Sex	.872	.771	.447
Age	.341	.718	.392
Locking duration	.252	.342	.427
MMO^†^	<.001	.639	.931
Joint pain at MMO^‡^	.634	.367	.159
Joint pain in biting^§^	.953	.974	.548
Disc shape	751	.693	.693
Bone change	.790	.676	.676
Synovitis	.186	.281	.281
Cartilage change	.260	.421	.421
Adhesion	.097	.521	.521
Disc perforation	.282	.878	.878

^†^MMO: Maximum mouth opening before arthrocentesis.

^‡^Joint pain at MMO: Joint pain at maximum mouth opening before arthrocentesis.

^§^Joint pain in biting: Joint pain in biting before arthrocentesis.
